# Editorial: Pediatric multiple sclerosis - from bench to bedside

**DOI:** 10.3389/fnins.2024.1381189

**Published:** 2024-04-09

**Authors:** Ethel Ciampi, Filipe Palavra

**Affiliations:** ^1^Neurology Department, Pontificia Universidad Catolica de Chile, Santiago, Chile; ^2^Neurology Service, Hospital Dr. Sótero del Río, Santiago, Chile; ^3^Center for Child Development—Neuropediatrics Unit, Hospital Pediátrico, Centro Hospitalar e Universitário de Coimbra, Coimbra, Portugal; ^4^Laboratory of Pharmacology and Experimental Therapeutics, Coimbra Institute for Clinical and Biomedical Research (iCBR), Faculty of Medicine, University of Coimbra, Coimbra, Portugal; ^5^Clinical Academic Center of Coimbra, Coimbra, Portugal

**Keywords:** multiple sclerosis, neuroimmunology, pediatric multiple sclerosis, neuroimmunological disease, neuroradiologic diagnosis

Multiple Sclerosis (MS) is a chronic inflammatory, demyelinating and neurodegenerative disease that is usually presented between 20 and 40 years of age. Nevertheless, ~5% of patients have their first clinical event before the age of 18 years, especially in patients younger than 10 years, presenting a challenge to physician with multiple differential diagnoses not usually included in classic adult-onset studies. Therapeutic decisions may also be challenging, particularly in assessing the risk-benefit balance in this highly active population, with good initial recovery, but with potentially irreversible accumulation of disability, despite disease-modifying therapy and symptomatic treatment.

Children also have inherent differences compared to adults that may be relevant to address, as their normal neurodevelopmental processes such as white matter myelination are being carried out. Therefore, novel magnetic resonance imaging (MRI) techniques that evaluate normal central nervous system (CNS) maturation, as well as the potential remyelination and repair, are needed. For example, motion correction for functional imaging that requires that the patient remains awake and still.

Filimonova et al. show their results using T1w to T2w signal intensity ratio (or T1w/T2w mapping), a neuroimaging method of measuring brain myelination, that requires only conventional T1w and T2w images, with no additional scanner time, as compared to diffusion-tensor imaging, magnetization transfer ratio, or myelin water fraction, that is very simple and is plausible for retrospective analyses using routine MRI protocols. They included 50 boys and 44 girls 0–23 months of age, showing a positive correlation of T1w/T2w with age, with a consistent spatiotemporal myelination pattern, with an excellent interrater agreement (ICC = 0.91). Soares et al. suggest a volume interpolation of motion outliers, using six motion parameters, as an alternative method to improve functional MRI (fMRI) analyses, mitigating the effects of head motion in task fMRI data. They included 17 early MS patients and 14 healthy controls, and although no differences were observed in terms of motion between patients and controls, this strategy may be particularly helpful in fMRI studies including children or patients with associated movement disorders. Faustino et al. performed an exploratory quantitative and radiomic characterization of 11 POMS patients including data from lesions, transitional tissue, and tissue without the presence of lesions on T2/FLAIR images. They selected 10 radiomic features with areas under the curve >85%, demonstrating the ability to discriminate between classes of interest.

Midaglia et al. present the particularly difficult case of a 9-year-old Morrocan girl who presented with a severe neuromyelitis optica spectrum disorder-like syndrome, with positive cerebrospinal fluid antibodies against myelin oligodendrocyte glycoprotein, weakly detected in serum, and positive oligoclonal bands, who later presented new typical MS MRI lesions, consistent with a final diagnosis of pediatric-onset MS (POMS). In the same line, Carvalho et al. present a challenging case series of 4 Caucasian patients diagnosed with MS between the ages of 4 and 9 (two boys and two girls), including the relevance of multidisciplinary discussion and agreement on the application of the McDonald Criteria. Disease-modifying therapy initiation was also difficult, as many parents may be prone to a “wait and see” approach, and they should be guided to an active clinical and radiological follow-up, also highlighting the opportunity to be included in clinical trials, mostly after the age of 10. In this context, it becomes particularly interesting the results from Palavra, Silva et al. exploring the clinical predictors of No Evidence of Disease Activity (NEDA3) after 1 year of follow-up in a cohort of 27 patients with POMS. They highlight the association of the absence of Epstein Barr Virus exposure with a higher probability of NEDA3 and show the pivotal importance of early and highly effective therapy in this group of patients, particularly with the use of natalizumab, supporting the importance of early treatment in preventing disability accumulation. In the second article, Palavra, Geria et al. also examine neutrophil/lymphocyte and monocyte/lymphocyte indices as potential predictors of relapse 1 year after diagnosis of pediatric multiple sclerosis. They found higher NLI and MLI values in relapsing patients compared to stable/inactive patients, although this difference was not statistically significant in this small sample size of 18 patients, further studies with a larger sample size could be relevant to determine the usefulness of this simple and easy to obtain biomarker. Finally, Stratton et al. report the interim guidelines for the assessment and treatment of pain in children with MS. They performed a modified Delphi study concluding the relevance of a variety of pain types, flare/remission periods that may affect their quality of life and differences compared to adult patients in the articulation of pain characteristics and functional impact. They propose child-friendly assessments, including functionality and activities of daily living, with a psychosocial and mental health screening and a holistic care team including pharmacological and non-pharmacological interventions.

In this Research Topic, we have tried to address several questions regarding clinical presentation, differential diagnosis, therapeutic strategies, and new imaging techniques that will certainly set the framework for a more stable bridge of translational neuroscience in pediatric multiple sclerosis from Bench to Bedside.

## Author contributions

EC: Writing – original draft, Writing – review & editing. FP: Writing – original draft, Writing – review & editing.

